# Metacognition and emotion regulation as treatment targets in binge eating disorder: a network analysis study

**DOI:** 10.1186/s40337-021-00376-x

**Published:** 2021-02-15

**Authors:** Matteo Aloi, Marianna Rania, Elvira Anna Carbone, Mariarita Caroleo, Giuseppina Calabrò, Paolo Zaffino, Giuseppe Nicolò, Antonino Carcione, Gianluca Lo Coco, Carlo Cosentino, Cristina Segura-Garcia

**Affiliations:** 1 Outpatient Unit for Clinical Research and Treatment of Eating Disorders, University Hospital “Mater Domini”, Catanzaro, Italy; 2 Department of Health Sciences, University “Magna Graecia” of Catanzaro, Catanzaro, Italy; 3 Department of Experimental and Clinical Medicine, School of Computer and Biomedical Engineering, University “Magna Graecia” of Catanzaro, Catanzaro, Italy; 4 Third Centre of Cognitive Psychotherapy – Italian School of Cognitive Psychotherapy (SICC), Rome, Italy; 5 Department of Psychology, Educational Science and Human Movement, University of Palermo, Palermo, Italy; 6 Department of Medical and Surgical Sciences, University “Magna Graecia” of Catanzaro, Catanzaro, Italy

**Keywords:** Binge eating disorder, Network analysis, Metacognition, Emotion dysregulation, Binge severity, Psychotherapy

## Abstract

**Background:**

This study aims to examine the underlying associations between eating, affective and metacognitive symptoms in patients with binge eating disorder (BED) through network analysis (NA) in order to identify key variables that may be considered the target for psychotherapeutic interventions.

**Methods:**

A total of 155 patients with BED completed measures of eating psychopathology, affective symptoms, emotion regulation and metacognition. A cross-sectional network was inferred by means of Gaussian Markov random field estimation using graphical LASSO and the extended Bayesian information criterion (EBIC-LASSO), and central symptoms of BED were identified by means of the strength centrality index.

**Results:**

Impaired self-monitoring metacognition and difficulties in impulse control emerged as the symptoms with the highest centrality. Conversely, eating and affective features were less central. The centrality stability coefficient of strength was above the recommended cut-off, thus indicating the stability of the network.

**Conclusions:**

According to the present NA findings, impaired self-monitoring metacognition and difficulties in impulse control are the central nodes in the psychopathological network of BED whereas eating symptoms appear marginal. If further studies with larger samples replicate these results, metacognition and impulse control could represent new targets of psychotherapeutic interventions in the treatment of BED. In light of this, metacognitive interpersonal therapy could be a promising aid in clinical practice to develop an effective treatment for BED.

**Supplementary Information:**

The online version contains supplementary material available at 10.1186/s40337-021-00376-x.

## Plain English summary

This study sought to examine the key symptoms for the psychotherapy of patients with binge eating disorder (BED). For this purpose, we applied a network analysis approach to examine the reciprocal association between clinical variables and how eating symptoms, metacognition, emotion regulation, depression and anxiety mutually interact. A total of 155 outpatients with BED completed measures related to their eating behaviour, affectivity, emotion regulation and metacognition. The central elements of BED were found to be impaired metacognition and difficulty in impulse control, whereas affective and eating symptoms appeared to be marginal. Therefore, metacognitive alterations and emotion dysregulation should be considered important targets for the psychotherapy of patients with BED.

## Background

Binge eating disorder (BED) is characterized by recurrent episodes of binge eating with a sense of loss of control over eating, accompanied by negative feelings [1]. To date, the guidelines recommend cognitive behavioural therapy (CBT) as the first-line treatment option for BED [2, 3]. Although CBT is quite effective in patients with BED, about 50% do not fully respond to treatment [4–6]. A possible explanation could be that only a small portion of patients with BED report the overvaluation of body shape and weight that forms the core of the CBT protocol [7]. Other treatments such as dialectical behaviour therapy [8, 9] and interpersonal psychotherapy [10, 11] have shown promising results but failed to bridge the efficacy gap in treating BED. In other words, the available data do not favour one treatment over the other.

New therapeutic approaches able to target the key elements of the complex psychopathology of BED are therefore a priority. Investigating the specific weight of each psychopathological dimension could help in developing more tailored psychological interventions for BED.

Network analysis (NA) emerged as a novel approach to conceptualize mental disorders [12]. According to this approach, symptoms of psychiatric disorders are distinct entities that can influence, maintain and/or interact with other symptoms [13]. Mental disorders can be characterized as complex systems in which symptoms are represented as distinct nodes, connected by edges that represent the strength (e.g. strong/weak correlations) and direction (e.g. positive/negative correlations) between pairs of symptoms. NA allows the identification of the central symptoms (i.e. when a node has many strong associations with other nodes and strong correlations with other nodes within the network) [14].

Development of the NA approach over the past decade has provided a theoretical framework that was adopted to identify the central symptoms of different psychiatric disorders, such as bipolar disorder [15], depression [16], obsessive compulsive disorder [17] and schizophrenia [18]. More recently, researchers in the field of eating disorders have applied NA to examine the symptoms of anorexia nervosa [19–22] and bulimia nervosa [23–25].

To date, only three studies [26–28] dealing with BED have used the NA approach. In the first investigation, overvaluation of shape and weight emerged as central symptoms of BED whereas behavioural symptoms (i.e. binge eating, restriction, secret eating) were less central [26]. The study by Solmi et al. revealed affective symptoms, interoceptive awareness, ineffectiveness, interpersonal functioning and drive for thinness as the central variables among patients with BED [27]. Finally, the third study showed that CBT provides high integration and connectivity of the psychopathology network in BED, suggesting an improved patient understanding of associations between binge eating and other symptoms [28].

However, no research has used NA to investigate the complex connections between the eating (i.e. binge eating and eating psychopathology), affective (i.e. anxiety and mood) and psychological (i.e. metacognition and emotion regulation) features of patients with BED.

Prior research evidenced a significant relationship among negative affect, difficulties with emotion regulation and binge eating symptoms [29–33]. For example, binge eating can be the result of a dysfunctional strategy to avoid interpersonal difficulties and negative emotions [34], especially in individuals who experience difficulties with regulating their emotional state [32]. However, the role of metacognition in BED has received less research attention. In the current study, we refer to metacognition as a psychological function that plays a key role in identifying mental states and ascribing them to oneself and others, reflecting and reasoning on mental states and, finally, using this information to manage interpersonal conflicts [35]. According to this model, metacognition is made up of different sub-functions that interact with each other and can be singularly impaired [35]. A previous study suggested that the severity of BED can worsen in relation to the impaired self-monitoring metacognition through the mediation of emotion dysregulation [36].

In the present study, we sought to extend the research on the clinical characteristics of BED by applying an NA model to provide an examination of the pathways that underlie eating symptoms and their relations to metacognition, emotion regulation and distress. These NA results may lead to more nuanced insights regarding the core targets for psychotherapeutic interventions. Given the explorative nature of our study, no a priori hypotheses were formulated.

## Methods

### Procedure

We performed a consecutive sampling of male and female patients attending the Outpatient Unit for Clinical Research and Treatment of Eating Disorders in Catanzaro (Italy). Patients were invited to participate in the present study if they met the following criteria: age 18–65 years; current diagnosis of BED according to the fifth edition of the *Diagnostic and Statistical Manual of Mental Disorders* (DSM-5) criteria; absence of current Axis I comorbid psychiatric disorders; and capable of answering self-report questionnaires and expressing valid consent.

Participants were deemed ineligible if they had: IQ < 70 [37]; drug dependence and/or abuse; severe mental illness that could interfere with clinical assessment (i.e. psychosis); history of chronic medical illness (i.e. chronic cardiovascular diseases) or neurological conditions (i.e. dementia) affecting cognitive functioning; other severe medical comorbidities (i.e. epilepsy); medical conditions that influenced eating/weight (i.e. diagnosis of diabetes mellitus); or a history of malignant disease.

Trained psychiatrists interviewed all participants using the Structured Clinical Interview for DSM-5 Disorders – Research Version [38] for diagnostic purposes and collected sociodemographic and clinical data. Researchers informed participants about the aims, procedures, anonymity and voluntary participation in this research. Participants gave their written informed consent to participate in accordance with the latest version of the Declaration of Helsinki [39] and the local ethical committee.

### Measures

The Eating Disorders Inventory-2 (EDI-2) [40, 41] is a self-report questionnaire made up of 91 items that evaluates the psychopathology and symptomatology of eating disorders. The EDI-2 provides 11 subscale scores and a global measure of eating disorder severity obtained from the sum of all the items (ranging from 0 to 273). Higher scores indicate more severe symptoms. Cronbach’s alpha for the total score in this study was good (.840).

The Binge Eating Scale (BES) [42] measures the severity of BED. It consists of 16 items that describe the behaviours, feelings and cognitions associated with binge eating. Total BES scores of < 17, 17–27 and > 27 indicate improbable, possible and probable BED, respectively. The internal consistency in this study was .880.

The Metacognition Self-Assessment Scale (MSAS) [43] is an 18-item five-point Likert-type (1 = never, 5 = almost always) self-report questionnaire that evaluates metacognitive functioning. The raw score ranges from 18 to 90 and lower scores indicate impaired self-evaluation of metacognitive function. Specifically, the MSAS measures four abilities of metacognition: monitoring, differentiation/decentration, integration and mastery. In this study, Cronbach’s alpha ranges from .820 to .840.

The Difficulties in Emotion Regulation Scale (DERS) [44] is a 36-item five-point Likert-type scale that assesses emotion dysregulation across six subscales: non-acceptance of emotions; difficulties in pursuing goals when having strong emotions; difficulties in controlling impulsive behaviours when experiencing negative emotions; lack of emotional awareness; limited access to emotion regulation strategies; and lack of emotional clarity. Higher scores indicate more problems in emotion regulation. In the current study, the internal consistency ranges from .870 to .895.

The Beck Depression Inventory II (BDI-II) [45] assesses depressive symptoms through 21 items on a Likert scale (0–3); scores of 0–9, 10–16, 17–29 and ≥ 30 indicate minimal, mild, moderate and severe depression, respectively. Cronbach’s alpha in the present research was .820.

The State-Trait Anxiety Inventory (STAI) consists of 20 items that assess state anxiety (STAI-St) and 20 items that measure trait anxiety (STAI-Tr) [46]. The present study only included the STAI-Tr for statistical purposes. Cronbach’s alpha was .795.

### Network estimation and accuracy

NA was performed using the R (version 3.6.2) *qgraph* and *bootnet* packages in accordance with Epskamp and colleagues [47].

The network has been inferred by means of Gaussian Markov random field estimation, applying ‘Least Absolute Shrinkage and Selection Operator’(LASSO) regularization to limit the number of spurious associations [48]. Moreover, the extended Bayesian information criterion (EBIC) [49], a tuning parameter that sets the degree of regularization/penalty applied to sparse correlations, was set to 0.20 in the current study (values between 0 and 0.5 are typically chosen). Network estimation was performed using the *estimateNetwork* routine of the *bootnet* package [50].

The centrality of a node is used to infer its influence, or structural importance, in the network. Three main indices estimate the centrality: *betweenness* (how a node influences the average path between other pairs of nodes); *closeness* (how a node is indirectly connected to the other nodes); and *strength* (how a node is directly connected to the other nodes). The centrality Plot function in *qgraph* was used to calculate indices of centrality.

According to the recommendations of Epskamp et al. [51], in order to assess the internal reliability of the network we calculated the correlation stability (CS) coefficient, which is the maximum proportion of the population that can be dropped so that the correlation between the re-calculated indices of the obtained networks and those of the original network is at least 0.7. It is recommended that the minimum cut-off to consider a network stable is 0.25 for *betweenness, closeness* and *strength* [51]. The CS coefficient was computed using case-drop bootstrapping (nboots = 2000). Then we estimated the accuracy of edge-weights by drawing bootstrapped confidence intervals calculated using non-parametric bootstrapping (nboots = 2000). Both for case-drop and non-parametric bootstrapping, network stability analyses were performed using the *bootnet* function in the *bootnet* package.

Visual inspection of the network reveals that thicker edges indicate stronger associations between symptoms, with positive associations typically illustrated in blue and negative associations typically represented in red.

## Results

### Sample characteristics

In total, 155 BED patients (86.5% females) aged 41.2 ± 13.2 years and with body mass index 37.9 ± 10.4 kg/m^2^_,_ took part in the current study. Table 1 displays the clinical characteristics of the sample.

**Table 1:** Clinical characteristics of the sample

	Mean	SD
EDI-2 Total	83.9	60.2
BES	23.4	9.3
STAI Trait	52.8	12.1
BDI	23.2	11.3
DERS		
Non acceptance	16.3	6.2
Goals	15.6	5.4
Impulse	15.8	6.2
Awareness	17.4	5.3
Strategies	22.1	8.8
Clarity	11.8	4.8
MSAS		
Self monitoring	18.4	5.0
Differentiation/Decentration	18.9	4.3
Mastery	16.5	4.2
Others monitoring	10.3	2.8

*EDI-2* Eating Disorder Inventory-2, *BES* Binge Eating Scale, *STAI* State and Trait Anxiety Inventory, *BDI* Beck Depression Inventory, *DERS* Difficulties in Emotion Regulation Scale, *MSAS* Metacognition Self-Assessment Scale, *SD* Standard Deviation

### Network analysis

**Fig. 1:**
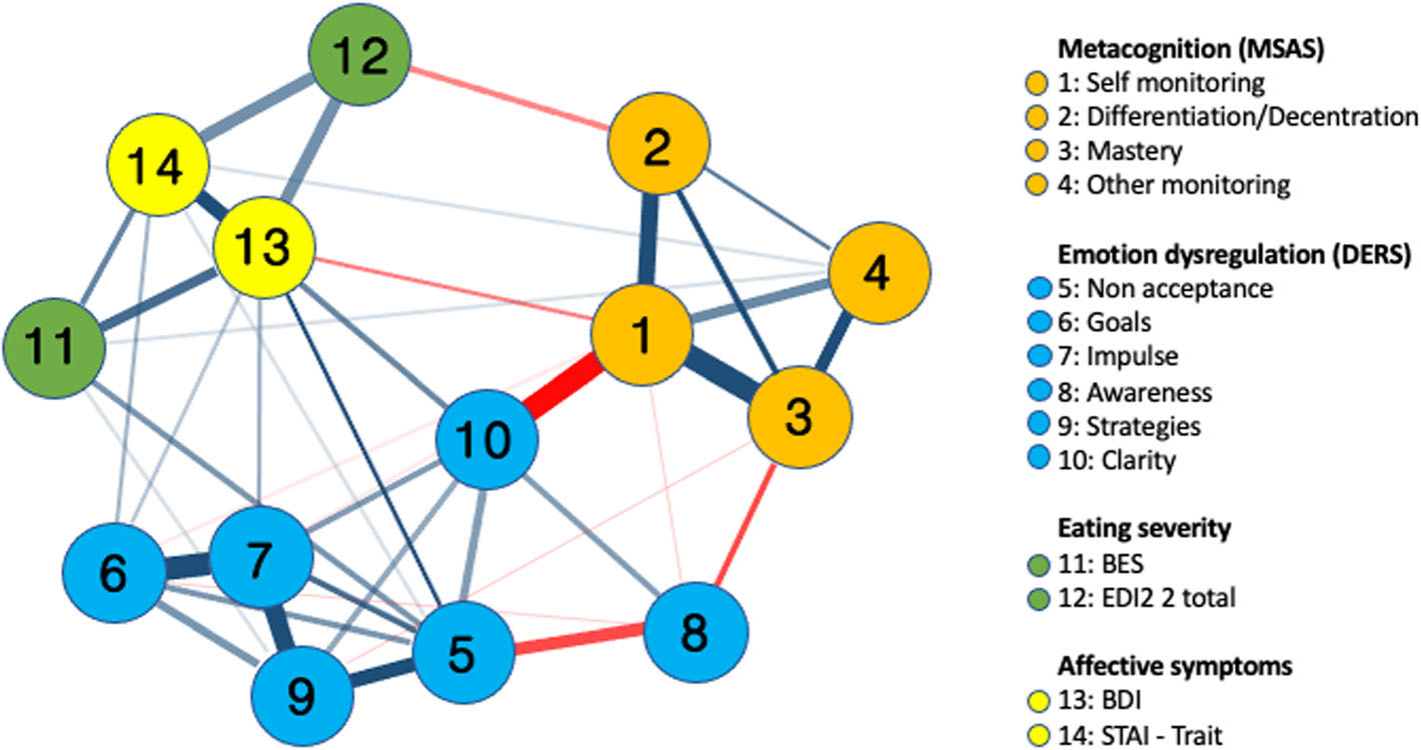
The network structure estimated from the graphical EBIC-LASSO in patients with binge eating disorder. Blue lines represent positive correlations, and red lines represent negative correlations. Thicker edges represent stronger correlations

**Fig. 2:**
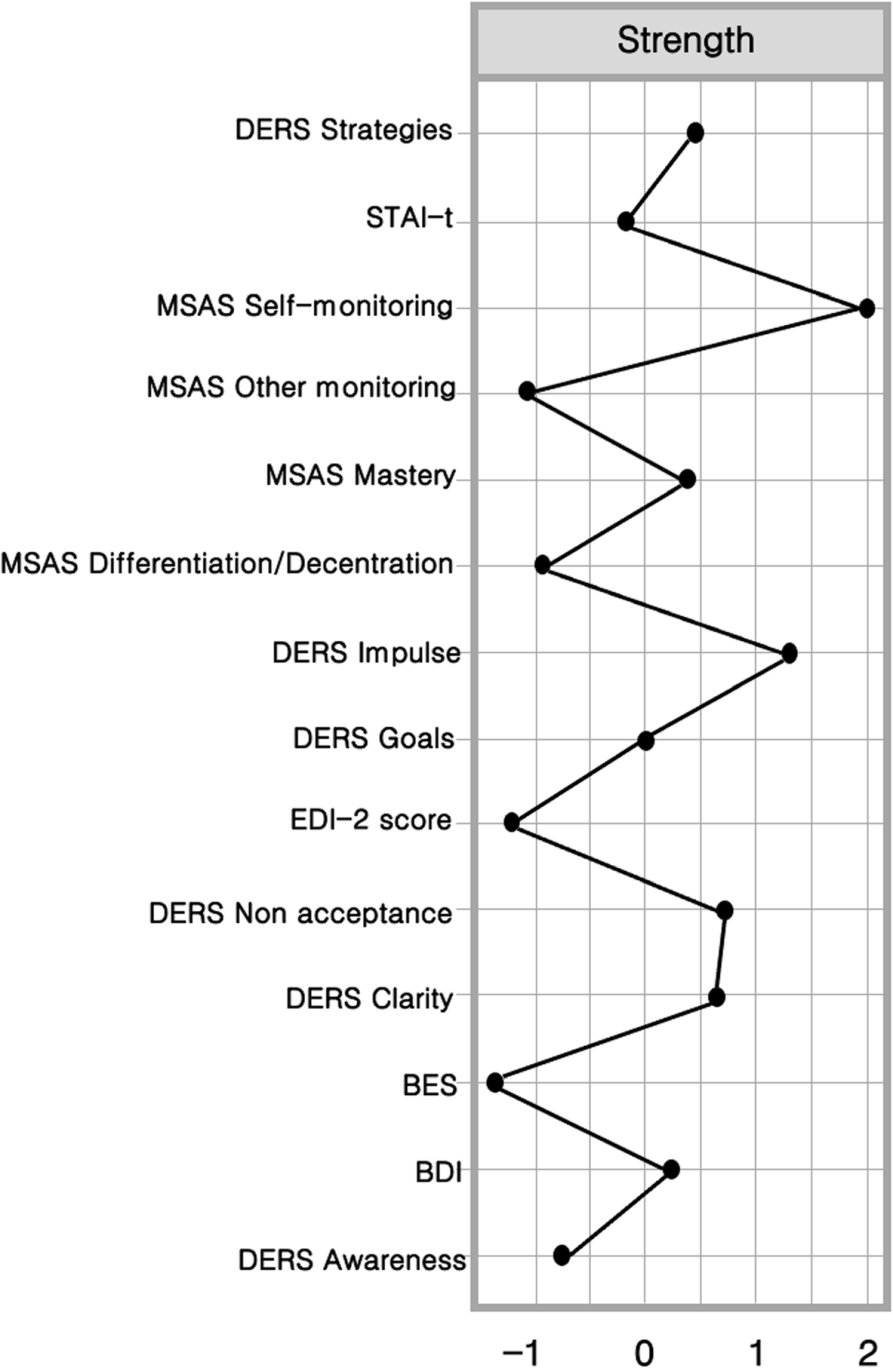
Plot of strength centrality index of the network for each node

Figure 1 illustrates the network of BED symptoms. Nodes belonging to each domain (i.e. eating symptoms, emotion dysregulation and metacognition) are generally associated and close to each other. There is a strong negative connection between self-monitoring and DERS-Clarity, and a strong positive connection among self-monitoring, differentiation and mastery. The associations between BED symptoms and depression, and between EDI-2 total score, depression and anxiety, are moderately strong. The psychopathological variables (BES, EDI-2 total score, STAI-Tr and BDI) and emotion regulation (DERS) are moderately connected. The BED symptom node (BES) has a direct connection with non-acceptance of emotions, whereas the depression node (BDI) connects both with difficulties in controlling impulsive behaviour and lack of emotional clarity. Figure 2 displays the strength centrality index of the variables included in the network. The CS coefficient is 0.301 for *strength*, which is above the recommended cut-off value (i.e. 0.25); however, the CS coefficients for *betweenness* and *closeness* are below 0.25. Therefore, we decided to choose the strength index as the main CS coefficient. This choice is not surprising because the interpretation of betweenness and closeness in networks is somewhat unclear [52] and the strength index is considered a more stable centrality index than betweenness and closeness [53]. Furthermore, because we aimed to understand the core symptoms to target using psychological treatment, we relied on the strength index because it exactly performs this function. Additional file 1 (Fig. S1) shows the accuracy of the CS indices.

The nodes with the highest strength centrality are MSAS-Self-monitoring (M = 1.98) and DERS-Impulse (M = 1.27) (Fig. 2). The strongest connections of MSAS-Self-monitoring are with MSAS-Mastery (0.352) and DERS-Clarity (− 0.350). The strongest connections of DERS-Impulse are with DERS-Goals (0.38) and DERS-Strategies (0.318). Additional file 2 (Fig. S2) reports the bootstrapped confidence intervals of the estimated edge-weights.

## Discussion

This is the first study to investigate the associations between eating (i.e. binge eating and eating psychopathology), affective (i.e. anxiety and depression) and psychological features (i.e. metacognition and emotion regulation) through the NA method among patients with BED.

Our results showed that impaired self-monitoring metacognition and difficulties in impulse control were the nodes with the highest centrality strength and thus the nodes most directly connected to the other nodes in the network [53]. According to the NA approach, activation of a node may cause the development of the connected symptoms; therefore, the most central nodes have been conceptualized as core symptoms [54]. Our findings suggest that impaired self-monitoring metacognition and difficulties in impulse control may be important clinical characteristics among patients with BED. Although the high centrality of a node may be the effect of connections with other symptoms [55] and a cross-sectional study cannot show causal associations, the metacognitive and emotion regulation dysfunctions may represent potential targets for treatment, therefore these outcome variables of BED warrant further research.

This finding is in line with our previous study where low self-monitoring led BED-obese patients to express the worsening of binge severity through emotion dysregulation [36]. Consistent with this hypothesis, other researchers found that difficulties in emotion recognition could play a key role in the development and maintenance of BED [56, 57].

Another important finding of the current NA was the strong correlation of the self-monitoring node with mastery strategies. According to metacognitive theory, a high level of self-monitoring allows the use of functional mastery strategies. In more detail, mastery is ‘*the ability to work through one’s representations and mental states, with a view to implementing effective action strategies, in order to accomplish cognitive tasks or cope with problematic mental states*’ [35, 58]. Thus, it could be inferred that enhancing metacognitive abilities leads to reduced dysfunctional strategies among patients with BED, who usually manage intense emotions with binges [8, 59].

It is worth noting that both dysfunctional eating (i.e. BES and EDI-2 total scores) and affective symptoms (i.e. BDI and STAI-Tr) were peripheral to the network structure of patients with BED, indicating that they had weaker connections to the rest of the network compared with other nodes. Regarding eating psychopathology, in the current study the lowest strength was found for the BES (M = − 1.39) and the EDI-2 total score (M = − 1.22). Notwithstanding the weak centrality of eating symptoms in the network structure, our findings suggest that the BES score is connected to non-acceptance of emotions, whereas the EDI-2 total score is connected to poor metacognitive ability to distance from one’s own thoughts and evaluate them critically. Overall, our results confirm recent literature data on NA in BED (that binge eating was not central to the psychopathology) [26, 28] but contrast with the typical approach to diagnosing BED (relying upon the presence of binge eating behaviours).

Consistent with the present findings, we could argue that the clinical constructs such as impaired self-monitoring, difficulties in impulse control and lack of emotional clarity could be the vulnerability factors of BED whereas the pathological eating behaviour (i.e. binge eating) itself seems to be the consequent behaviour. This observation is in line with recent literature that investigated predisposing and precipitating factors in BED [32, 60, 61].

Furthermore, depressive and anxious symptoms were not central nodes in our network model whereas they had high centrality in Solmi and colleagues’ model [27]. This discrepancy could be due to the use of different psychometric instruments. Solmi and colleagues used the Symptom Checklist-90 (SCL-90), which is not so specific and only takes into consideration the prior week; instead, the BDI-II and the STAI-Tr are more specific for diagnostic purposes and consider a longer temporal range of assessment (i.e. 2 weeks for BDI following the DSM-5 temporal criterion for major depressive episode; ‘usually feeling’ for STAI-Tr). Therefore, their study could have overestimated the weight of anxious and depressive symptoms in BED.

The present results should be read in light of some limitations. First, the sample size is smaller than in other studies that used NA in BED. Nevertheless, according to the recommendations of Levinson and colleagues [62] on the use of NA in the field of eating disorder (‘*to date, the best recommendation is to use the largest sample size possible and make sure that your network is stable*’), our model was demonstrated to be stable. Second, it was not possible to evaluate the differences in NA according to gender; however, a recent NA study among patients with eating disorders showed more similarities than differences between men and women [63]. Finally, the cross-sectional design does not allow the investigation of causality in the associations between dimensions, therefore future longitudinal research could explore whether psychotherapeutic interventions that target metacognitive and impulsive dimensions may be more effective in treating BED.

## Conclusions

The current study suggests a link between reduced ability to identify and describe mental states and the lack of emotion awareness and clarity among patients with BED. Moreover, according to the present NA findings, impaired self-monitoring metacognition and difficulties in impulse control are the central nodes in the psychopathological network of BED, whereas eating symptoms seem to be marginal.

These results could lead to a change in the current conceptualization of BED and the consideration of new targets of psychotherapeutic interventions, if confirmed in larger samples. Also, approaches focused on the improvement of metacognitive dysfunctions could be considered. With this aim, metacognitive interpersonal therapy [64] could be a promising aid in clinical practice to develop an effective treatment for BED.

## Supplementary Information

**Additional file 1: Figure S1**. Results of case-dropping subset bootstrap procedure to assess stability of network centrality indices. Average correlations between centrality indices of networks sampled with persons dropped and the original sample.

**Additional file 2: Figure S2.** Bootstrapped confidence intervals (#boots = 2000) for estimated edge-weights of BED patients.

## Data Availability

The datasets used and/or analysed during the current study are available from the corresponding author on reasonable request.

## Abbreviations

BED: Binge eating disorder
BES: Binge eating scale
BDI-II: Beck Depression Inventory II
CBT: Cognitive behavioral therapy
CS: Correlation stability
DBT: Dialectical behavioral therapy
DERS: Difficulties in Emotion Regulation Scale
DSM-5: Diagnostic and Statistical Manual of Mental Disorders – 5
EBIC: Extended Bayesian Information Criterion
ED: Eating disorder
EDI-2: Eating Disorders Inventory-2
IPT: Interpersonal psychotherapy
LASSO: Least Absolute Shrinkage and Selection Operator
MIT: Metacognitive Interpersonal Therapy
MSAS: Metacognition Self-Assessment Scale
NA: Network analysis
SCL-90: Symptom checklist – 90
STAI-Tr: State-Trait Anxiety Inventory-Trait

## References

[1] APA AAP (2013). Diagnostic and statistical manual of mental disorders (DSM-5®).

[2] Hay P, Chinn D, Forbes D, Madden S, Newton R, Sugenor L (2014). Royal Australian and new Zealand College of Psychiatrists clinical practice guidelines for the treatment of eating disorders. Aust New Zeal J Psychiatry.

[3] National Institute for Health and Care Excellence (2017). Eating disorders: Recognition and treatment.

[4] Grilo CM (2017). Psychological and behavioral treatments for binge-eating disorder. J Clin Psychiatry.

[5] Hay P (2013). A systematic review of evidence for psychological treatments in eating disorders: 2005-2012. Int J Eat Disord.

[6] Brownley KA, Berkman ND, Peat CM, Lohr KN, Cullen KE, Bann CM (2016). Binge-Eating Disorder in Adults. Ann Intern Med.

[7] Grilo CM (2013). Why no cognitive body image feature such as overvaluation of shape/weight in the binge eating disorder diagnosis?. Int J Eat Disord.

[8] Chen EY, Cacioppo J, Fettich K, Gallop R, McCloskey MS, Olino T (2017). An adaptive randomized trial of dialectical behavior therapy and cognitive behavior therapy for binge-eating. Psychol Med.

[9] Lammers MW, Vroling MS, Crosby RD, van Strien T (2020). Dialectical behavior therapy adapted for binge eating compared to cognitive behavior therapy in obese adults with binge eating disorder: a controlled study. J Eat Disord.

[10] Linardon J (2018). Rates of abstinence following psychological or behavioral treatments for binge-eating disorder: meta-analysis. Int J Eat Disord.

[11] Hilbert A, Hildebrandt T, Agras WS, Wilfley DE, Wilson GT (2015). Rapid response in psychological treatments for binge eating disorder. J Consult Clin Psychol.

[12] Robinaugh DJ, Hoekstra RHA, Toner ER, Borsboom D (2020). The network approach to psychopathology: a review of the literature 2008–2018 and an agenda for future research. Psychol Med.

[13] McNally RJ (2016). Can network analysis transform psychopathology?. Behav Res Ther.

[14] Freeman LC (1978). Centrality in social networks conceptual clarification. Soc Networks.

[15] Scott J, Bellivier F, Manchia M, Schulze T, Alda M, Etain B (2020). Can network analysis shed light on predictors of lithium response in bipolar I disorder?. Acta Psychiatr Scand.

[16] Corponi F, Anmella G, Verdolini N, Pacchiarotti I, Samalin L, Popovic D (2020). Symptom networks in acute depression across bipolar and major depressive disorders: A network analysis on a large, international, observational study. Eur Neuropsychopharmacol.

[17] Olatunji BO, Christian C, Brosof L, Tolin DF, Levinson CA (2019). What is at the core of OCD? A network analysis of selected obsessive-compulsive symptoms and beliefs. J Affect Disord.

[18] Galderisi S, Rucci P, Mucci A, Rossi A, Rocca P, Bertolino A (2020). The interplay among psychopathology, personal resources, context-related factors and real-life functioning in schizophrenia: stability in relationships after 4 years and differences in network structure between recovered and non-recovered patients. World Psychiatry.

[19] Calugi S, Sartirana M, Misconel A, Boglioli C, Dalle GR (2020). Eating disorder psychopathology in adults and adolescents with anorexia nervosa: a network approach. Int J Eat Disord.

[20] Cascino G, Castellini G, Stanghellini G, Ricca V, Cassioli E, Ruzzi V (2019). The Role of the Embodiment Disturbance in the Anorexia Nervosa Psychopathology: A Network Analysis Study. Brain Sci.

[21] Monteleone AM, Cascino G, Pellegrino F, Ruzzi V, Patriciello G, Marone L (2019). The association between childhood maltreatment and eating disorder psychopathology: a mixed-model investigation. Eur Psychiatry.

[22] Kerr-Gaffney J, Halls D, Harrison A, Tchanturia K. Exploring Relationships Between Autism Spectrum Disorder Symptoms and Eating Disorder Symptoms in Adults With Anorexia Nervosa: A Network Approach. Front Psychiatry. 2020;11 Available from: https://www.frontiersin.org/article/10.3389/fpsyt.2020.00401/full. 10.3389/fpsyt.2020.00401 PMC7235355 32477185

[23] Levinson CA, Zerwas S, Calebs B, Forbush K, Kordy H, Watson H (2017). The core symptoms of bulimia nervosa, anxiety, and depression: a network analysis. J Abnorm Psychol.

[24] Monteleone AM, Corsi E, Cascino G, Ruzzi V, Ricca V, Ashworth R, et al. The Interaction Between Mentalizing, Empathy and Symptoms in People with Eating Disorders: A Network Analysis Integrating Experimentally Induced and Self-report Measures. Cognit Ther Res. 2020; Available from: http://link.springer.com/10.1007/s10608-020-10126-z.

[25] Brown TA, Vanzhula IA, Reilly EE, Levinson CA, Berner LA, Krueger A (2020). Body mistrust bridges interoceptive awareness and eating disorder symptoms. J Abnorm Psychol.

[26] Wang SB, Jones PJ, Dreier M, Elliott H, Grilo CM (2019). Core psychopathology of treatment-seeking patients with binge-eating disorder: a network analysis investigation. Psychol Med.

[27] Solmi M, Collantoni E, Meneguzzo P, Degortes D, Tenconi E, Favaro A (2018). Network analysis of specific psychopathology and psychiatric symptoms in patients with eating disorders. Int J Eat Disord.

[28] Hilbert A, Herpertz S, Zipfel S, Tuschen-Caffier B, Friederich H-C, Mayr A, et al. Psychopathological Networks in Cognitive-Behavioral Treatments for Binge-Eating Disorder. Psychother Psychosom. 2020:1–7 Available from: https://www.karger.com/Article/FullText/509458. 10.1159/000509458 32694245

[29] Aloi M, Rania M, Caroleo M, Carbone EA, Fazia G, Calabrò G (2020). How are early maladaptive schemas and DSM-5 personality traits associated with the severity of binge eating?. J Clin Psychol.

[30] Araujo DMR, da Silva Santos GF, Nardi AE (2010). Binge eating disorder and depression: a systematic review. World J Biol Psychiatry.

[31] Caroleo M, Primerano A, Rania M, Aloi M, Pugliese V, Magliocco F, et al. A real world study on the genetic, cognitive and psychopathological differences of obese patients clustered according to eating behaviours. Eur Psychiatry. 2018;48:58–64. 10.1016/j.eurpsy.2017.11.009. Epub 2018 Jan 10. PMID: 29331600. 10.1016/j.eurpsy.2017.11.009 29331600

[32] Burton AL, Abbott MJ. Processes and pathways to binge eating: development of an integrated cognitive and behavioural model of binge eating. J Eat Disord. 2019;7:–18 Available from: https://jeatdisord.biomedcentral.com/articles/10.1186/s40337-019-0248-0. 10.1186/s40337-019-0248-0 PMC6554957 31183111

[33] Leehr EJ, Krohmer K, Schag K, Dresler T, Zipfel S, Giel KE (2015). Emotion regulation model in binge eating disorder and obesity - a systematic review. Neurosci Biobehav Rev.

[34] Lo Coco G, Sutton R, Tasca GA, Salerno L, Oieni V, Compare A (2016). Does the interpersonal model generalize to obesity without binge eating?. Eur Eat Disord Rev.

[35] Semerari A, Carcione A, Dimaggio G, Falcone M, Nicolò G, Procacci M (2003). How to evaluate metacognitive functioning in psychotherapy? The metacognition assessment scale and its applications. Clin Psychol Psychother.

[36] Aloi M, Rania M, Carbone EA, Calabrò G, Caroleo M, Carcione A (2020). The role of self-monitoring metacognition sub-function and negative urgency related to binge severity. Eur Eat Disord Rev.

[37] Wechsler D (2013). WAIS-iv. Wechsler Adult Intelligence Scale.

[38] First MB, Williams JBW, Karg RS, Spitzer RL (2015). User’s guide for the structured clinical interview for DSM-5 disorders, research version (SCID-5-RV).

[39] World Medical Association (2013). Declaration of Helsinki. JAMA.

[40] Garner DM (1991). Eating disorder Inventory-2. Professional manual.

[41] Segura-García C, Aloi M, Rania M, Ciambrone P, Palmieri A, Pugliese V (2015). Ability of EDI-2 and EDI-3 to correctly identify patients and subjects at risk for eating disorders. Eat Behav.

[42] Ricca V, Mannucci E, Moretti S, Di Bernardo M, Zucchi T, Cabras PL (2000). Screening for binge eating disorder in obese outpatients. Compr Psychiatry.

[43] Pedone R, Semerari A, Riccardi I, Procacci M, Nicolò G, Carcione A (2017). Development of a self-report measure of metacognition: the metacognition self-assessment scale (MSAS). Instrument description and factor structure. Clin Neuropsychiatry J Treat Eval.

[44] Giromini L, Velotti P, de Campora G, Bonalume L, Cesare ZG (2012). Cultural adaptation of the difficulties in emotion regulation scale: reliability and validity of an Italian version. J Clin Psychol.

[45] Ghisi M, Flebus GB, Montano A, Sanavio E, Sica C (2006). Beck depression inventory-II. Manuale italiano.

[46] Pedrabissi L, Santinello M (1989). Inventario per l’Ansia di ‘Stato’ e di ‘Tratto’: Nuova Versione Italiana dello STAI —Forma Y: Manuale.

[47] Epskamp S, Cramer AOJ, Waldorp LJ, Schmittmann VD, Borsboom D. qgraph : Network Visualizations of Relationships in Psychometric Data. J Stat Softw. 2012;48 Available from: http://www.jstatsoft.org/v48/i04/.

[48] Friedman J, Hastie T, Tibshirani R (2014). Glasso: graphical Lasso-estimation of Gaussian graphical models; R package version 1.

[49] Chen J, Chen Z (2008). Extended Bayesian information criteria for model selection with large model spaces. Biometrika.

[50] Epskamp S, Maris G, Waldorp L, Borsboom D, Irwing P, Hughes D, Booth T (2016). Network psychometrics. Handb Psychom.

[51] Epskamp S, Borsboom D, Fried EI (2018). Estimating psychological networks and their accuracy: a tutorial paper. Behav Res Methods.

[52] Forbes MK, Wright AGC, Markon KE, Krueger RF (2017). Evidence that psychopathology symptom networks have limited replicability. J Abnorm Psychol.

[53] Epskamp S, Fried EI (2018). A tutorial on regularized partial correlation networks. Psychol Methods.

[54] Fried EI, Cramer AOJ (2017). Moving forward: challenges and directions for psychopathological network theory and methodology. Perspect Psychol Sci.

[55] Forbes MK, Wright AGC, Markon KE, Krueger RF (2019). The network approach to psychopathology: promise versus reality. World Psychiatry.

[56] Prefit A-B, Cândea DM, Szentagotai-Tătar A (2019). Emotion regulation across eating pathology: A meta-analysis. Appetite.

[57] Westwood H, Kerr-Gaffney J, Stahl D, Tchanturia K (2017). Alexithymia in eating disorders: systematic review and meta-analyses of studies using the Toronto alexithymia scale. J Psychosom Res.

[58] Carcione A, Nicolò G, Pedone R, Popolo R, Conti L, Fiore D (2011). Metacognitive mastery dysfunctions in personality disorder psychotherapy. Psychiatry Res.

[59] Safer DL, Telch CF, Chen EY (2009). Dialectical behavior therapy for binge eating and bulimia.

[60] Dingemans A, Danner U, Parks M (2017). Emotion Regulation in Binge Eating Disorder: A Review. Nutrients.

[61] Treasure J, Duarte TA, Schmidt U (2020). Eating disorders. Lancet.

[62] Levinson CA, Vanzhula IA, Brosof LC, Forbush K. Network Analysis as an Alternative Approach to Conceptualizing Eating Disorders: Implications for Research and Treatment. Curr Psychiatry Rep [Internet]. 2018;20:67. Available from: http://link.springer.com/10.1007/s11920-018-0930-y 10.1007/s11920-018-0930-y 30079431

[63] Perko VL, Forbush KT, Siew CSQ, Tregarthen JP (2019). Application of network analysis to investigate sex differences in interactive systems of eating-disorder psychopathology. Int J Eat Disord.

[64] Carcione A, Riccardi I, Bilotta E, Leone L, Pedone R, Conti L, et al. Metacognition as a Predictor of Improvements in Personality Disorders. Front Psychol. 2019;10 Available from: https://www.frontiersin.org/article/10.3389/fpsyg.2019.00170/full. 10.3389/fpsyg.2019.00170 PMC6375846 30800084

