# Supplementary figures and images for: Metacognition and emotion regulation as treatment targets in binge eating disorder: a network analysis study
Source: J Eat Disord. 2021 Feb 15;9:22. doi: 10.1186/s40337-021-00376-x (PMC7885411; doi:10.1186/s40337-021-00376-x)

| Figure S1. |
| --- |
| 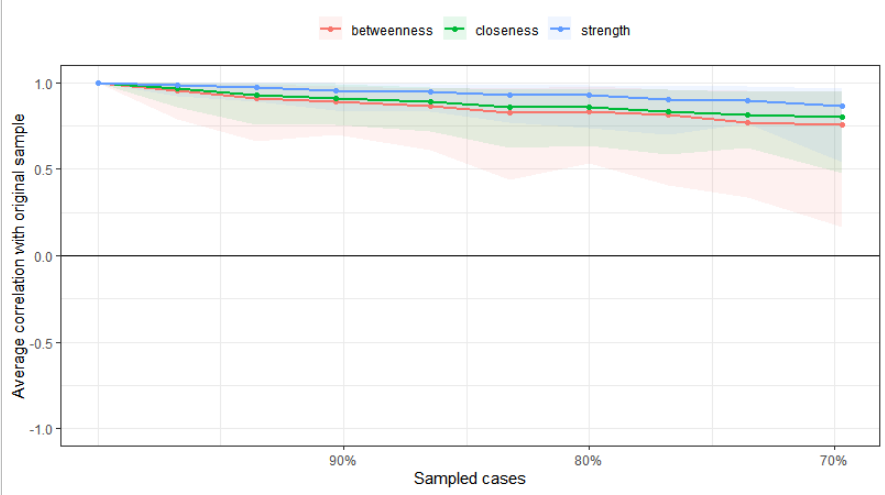 |

Supplement: Supplementary file 1 — Additional file 1: Figure S1. Results of case-dropping subset bootstrap procedure to assess stability of network centrality indices. Average correlations between centrality indices of networks sampled with persons dropped and the original sample. [file 40337_2021_376_MOESM1_ESM.docx]

| Figure S2. |
| --- |
| 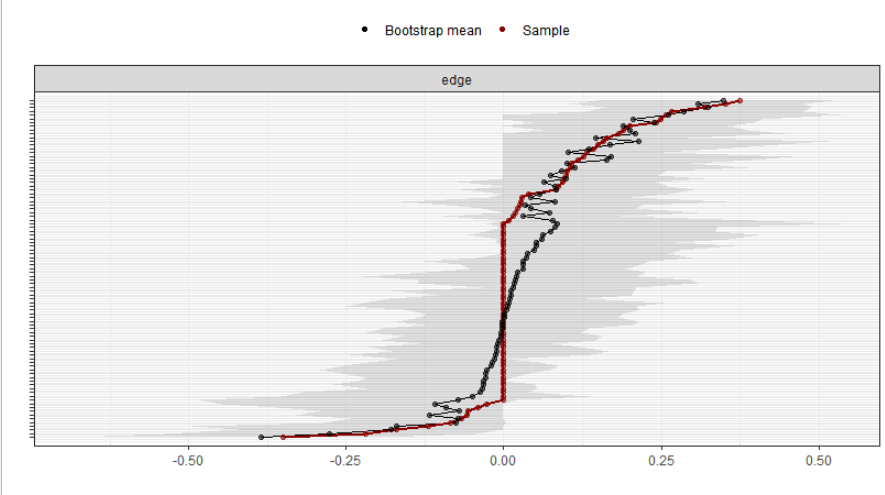 |

Supplement: Supplementary file 2 — Additional file 2: Figure S2. Bootstrapped confidence intervals (#boots = 2000) for estimated edge-weights of BED patients. [file 40337_2021_376_MOESM2_ESM.docx]
